# What is the minimum adequate busulfan dose for patients with sickle cell disease undergoing reduced intensity conditioning with fludarabine, busulfan, and anti‐thymocyte globulin?

**DOI:** 10.1002/jha2.218

**Published:** 2021-05-07

**Authors:** Naif I. AlJohani, Amir Ahmed Toor

**Affiliations:** ^1^ Adult Hematology and Bone Marrow Transplant Section Oncology Department King Faisal Specialist Hospital and Research Centre Jeddah Saudi Arabia; ^2^ Department of Internal Medicine BMT program Massey Cancer Center Virginia Commonwealth University Richmond Virginia USA

**Keywords:** busulfan, chimerism, hematopoietic stem cell transplantation, sickle cell anemia

## Abstract

Fludarabine busulfan anti‐thymocyte globulin is a common conditioning chemotherapy with reduced toxicity used for transplantation in sickle cell disease (SCD). The dose of busulfan used in this protocol is variable across studies and centers. The minimum dose that maintains long‐term donor chimerism is not well established. We hypothesized that a lower, less‐toxic dose could be used to maintain adequate long‐lasting chimeras, which might allow for the inclusion of older or comorbid patients with this disease. In our retrospective study of 11 patients, 8–9.6 mg/kg was adequate to maintain chimerism in six patients. A 6 mg/kg dose resulted in transplant rejection in two patients. This suggests that 0.8 mg/kg IV busulfan every 6 h for 8–12 doses (total 8–9.6 mg/kg) is the minimum adequate busulfan dose required to maintain long‐lasting chimeras, facilitating the successful withdrawal of immunosuppression in SCD patients who receive this protocol.

## 1 INTRODUCTION

Sickle cell disease (SCD) is a globally prevalent disease, with only 50% of patients surviving beyond 50 years of age. Treatment for the disease includes simple or exchange transfusion and hydroxycarbamide administration. However, the only available curative treatment options are hematopoietic stem cell transplantation (HSCT) and gene therapy [1]. Historically, HSCT has seldom been used because of the limited number of donors, as well as its potential safety risks. Meanwhile, there has been rapid progress in the development of reduced‐intensity conditioning regimens for HLA‐matched, related, and unrelated donor transplantations, with an average survival rate of more than 90% and average event‐free survival of more than 85% [2, 3]. These conditioning regimens have dramatically increased the number of transplantations performed lately.

The establishment of adequate chimerism is of utmost importance to cure SCD. A previous study showed that low‐level donor chimerism (11%) in whole blood is sufficient to render patients free of the clinical manifestations of SCD [4]; however, lineage‐specific chimerism was not investigated in that study. Another study by Fitzhugh et al, using a non‐myeloablative conditioning regimen, showed that as little as 20% donor myeloid chimerism is sufficient to reverse the sickle cell phenotype after allogeneic HSCT [5].

In the recent STRIDE clinical trial published in the American Journal of Hematology, 22 patients (median age, 22 years; age range, 17–36 years) from eight centers were administered a reduced‐intensity conditioning regimen of intravenous busulfan (13.2 mg/kg) and fludarabine (175 mg/m^2^) with rabbit anti‐thymocyte globulin (ATG; 6 mg/kg; Thymoglobulin; Sanofi‐Aventis), cyclosporine or tacrolimus, and methotrexate for prophylaxis against graft‐versus‐host disease (GVHD) [3]. One patient died of intracranial hemorrhage secondary to posterior, reversible, encephalopathy syndrome, and another patient died of GVHD. Full donor myeloid chimerism, along with mixed T‐cell chimerism, was observed (average: 82% donor on day +365) in 21 of 22 patients.

The minimum dose of busulfan needed to maintain sustained donor chimerism has not yet been established. We hypothesized that reduced‐intensity conditioning with doses ranging from 6 to 9.6 mg might be able to maintain the long‐term reversal of the sickle cell phenotype by sustaining donor myeloid chimerism at greater than 90%, with acceptable, stable, mixed hematopoietic chimerism over long periods, thereby allowing for safe immunosuppression withdrawal. We used this regimen in patients with SCD requiring transplantation as per the National Institutes of Health guidelines [6].

## 2 MATERIALS AND METHODS

The aforementioned protocol, with minor modifications, has been adopted at our Center since 2018. We administered conditioning chemotherapy that consisted of busulfan 0.8 mg/kg IV every 6 h for 8–12 doses from day −6 to day −3, fludarabine 30 mg/m^2^/day for 5 days from day −7 to −3, and 1.5 mg/kg of rabbit ATG from day −5 to day −2 (total dose of 6 mg). GVHD prophylaxis comprised cyclosporine with a target range of 200–400 ng/ml in addition to 15 mg/m^2^ intra‐venous methotrexate on day +1, as well as 10 mg/m^2^ on days +3 and +6. Immunosuppression was continued in outpatient settings until 9 months post‐transplant. All donors were HLA‐matched related donors, and bone marrow was used as the stem cell source. All patients received standard supportive care with anti‐*Candida* fluconazole prophylaxis, levofloxacin for neutropenia prophylaxis, and acyclovir for herpes simplex virus and varicella zoster virus prophylaxis. Weekly cytomegalovirus virus monitoring was performed for all patients until 100 days and then at longer intervals if applicable. During the peri‐transplant period, blood transfusion was given to maintain hemoglobin above 8 g/dl, and platelets were transfused to maintain platelet counts above 50,000/μl. In total, 11 patients underwent transplantation. The median age at the time of transplant was 24 years; six patients were male and five were female. All patients had SS disease with baseline Hb SS of 80%–90%. All patients had more than three episodes of vaso‐occlusive crisis in the 2 years prior to transplant, five had acute chest syndrome, and two required intensive care unit admission. One patient had a stroke and moyamoya syndrome and received pial synangiosis revascularization surgery prior to transplant. All patients were on hydroxyurea and were either non‐responsive or intolerant. Two patients had resolved hepatitis b and one had hepatitis c virus infection, which was treated prior to transplant. The choice of the total number of doses of busulfan was the clinician's prerogative, based on certain patient factors that might increase the risk of veno‐occlusive disease. Four patients received a total of 6.4 mg/kg of busulfan, five received 9.6 mg/kg, one received 8 mg/kg, and one received 12.8 mg/kg of busulfan. All patients are currently alive, except one, aged 36 years, who died 3 months post‐bone marrow transplantation (HSCT) because of community‐acquired viral pneumonia. Two categories of busulfan dosing regimens (6.4 mg [8 doses] (*N* = 4) and 8–9.6 mg [10–12 doses] (*N* = 5)) were used for data analysis. Data analysis and calculation of the coefficient of variation (CV) were performed using R software.

## 3 RESULTS

### 3.1 Lymphoid and myeloid chimerism at different doses of busulfan across the sampling time points

**FIGURE 1:**
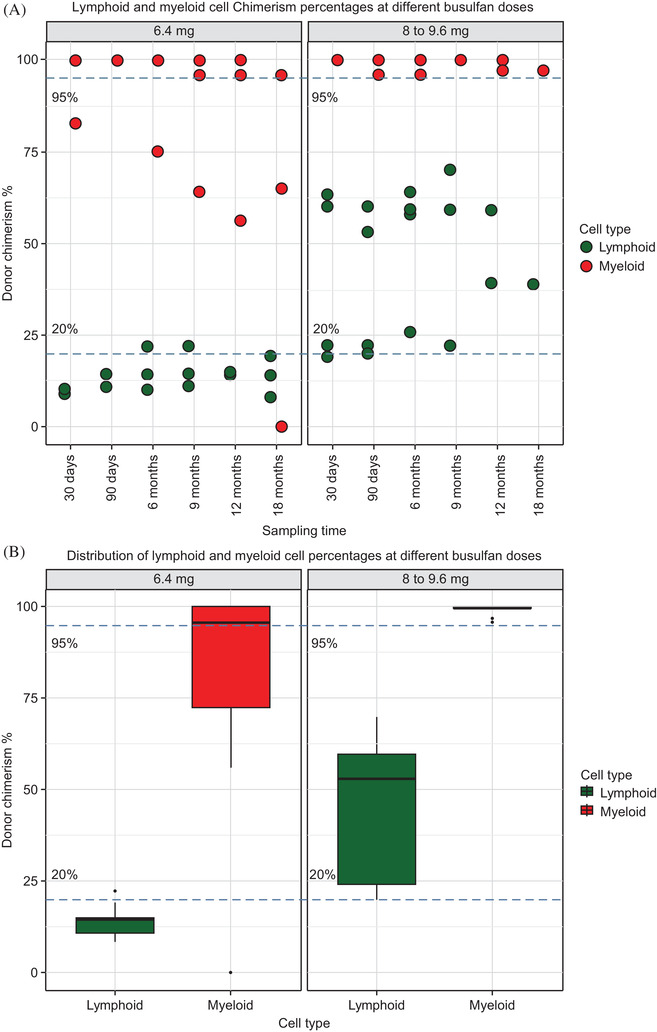
(A) Lymphoid and myeloid cell chimerism with different busulfan doses. (B) Distribution of lymphoid and myeloid cell percentages with different busulfan doses

Blood samples for chimerism measurements were collected at 1, 3, 6, 9, 12, and 18 months post‐transplantation. The mean lymphoid chimerism for the 6.4 mg and 8–9.6 mg categories was 13.8% (standard deviation [SD] 4.35%) and 45.6% (SD 18.19%), respectively. For myeloid chimerism, the means for the 6.4 mg and 8–9.6 mg categories were 83.19% (SD 26.95%) and 99.26% (SD 1.48%), respectively. There was significant variation in myeloid chimerism at low doses of busulfan, with greater stability seen at higher doses (8–9.6 mg). The variation in the level of chimerism achieved was higher in lymphoid cells than in myeloid cells, at both low and high doses. This variation was confirmed using the CV from the data analysis (Figure 1, Panel A). A Wilcoxon signed‐rank test was performed to check the effect of the two dose categories of busulfan on myeloid and lymphoid cells using the donor cell population percentages of the patient data at different time points. This test was chosen due to the repeated nature of the data collection and the non‐normality of the data. The normality of the data was checked using the Shapiro‐Wilk test. Wilcoxon test results showed a significant difference between the myeloid and lymphoid donor cell population percentages at both the lower and higher concentrations (*p* < 0.001). Myeloid cells showed a decrease in CV (0.02%) when the dose was increased (9.6 mg/kg), which suggested stability in the values. Moreover, an increase in the dose of busulfan from 6.4 to 9.6 mg/kg increased the stability of donor myeloid engraftment but not that of lymphoid cells (Figure 1, Panel B).

### 3.2 Clinical outcomes

Engraftment occurred in all patients. The median time for neutrophil engraftment was 22 days, and the median time for platelet engraftment was 20 days. The mean Hb levels at 3 and 12 months post‐transplant were 116 g/l and 118 g/l, respectively, for the 6 mg busulfan category, compared to 116 g/l and 120 g/l, respectively, for the 8–9.6 mg busulfan category. At the median follow‐up times of 1 year, 10 months, and 15 days, none of the patients who received 8–9.6 mg/kg busulfan rejected their transplants (*N* = 5), with stable myeloid and lymphoid chimerism, including two patients for whom immunosuppressive therapy was discontinued. No patient developed GVHD or required blood transfusion.

In contrast, two of the four patients who received 6.4 mg/kg of busulfan rejected their transplants, with a complete loss of donor chimerism and reversion to the sickle cell phenotype. One of them rejected the transplant at 6 months post‐HSCT while on immunosuppressive therapy but underwent successful re‐transplantation using a non‐myeloablative regimen with alemtuzumab/total body irradiation (0.03 mg/kg on day −7, 0.1 mg/kg on day −6, and 0.3 mg/kg/day from days −5 to −3; total body irradiation was given as a single dose of 300 cGy on day −2). This patient showed 100% myeloid and lymphoid donor chimerism 11 months after the second HSCT. The second patient rejected the transplant 1 year and 4 months post‐HSCT, 4 months after stopping immunosuppression, which typically occurs at 9–12 months post‐HSCT. This patient is currently awaiting re‐transplantation.

## 4 CONCLUSION

In conclusion, our clinical observation of HSCT for SCD revealed that 8–9.6 mg/kg of intravenous busulfan maintained nearly complete myeloid chimerism and stable mixed lymphoid chimerism with a median follow‐up time of 1 year and 4 months, allowing for safe immunosuppressive therapy withdrawal. There was a high risk of declining chimerism and graft rejection with a busulfan dose of 6 mg/kg or less. This study had a small sample size; therefore, further investigations for the optimization of busulfan dosing in SCD patients after HSCT are recommended.

## CONFLICT OF INTEREST

The authors declare that there is no conflict of interest that could be perceived as prejudicing the impartiality of the research reported.

## ETHICS APPROVAL

This study was approved by the Hospital Institutional Review Board at King Faisal Specialist Hospital and Research Center, Jeddah, Saudi Arabia.

## PATIENT CONSENT STATEMENT

Written informed consent was obtained from all patients.

## Data Availability

The data that support the findings of this study are available upon request from the corresponding author. The data are not publicly available due to privacy or ethical restrictions.
